# Pharmacokinetics and metabolism of ifosfamide in relation to DNA damage assessed by the COMET assay in children with cancer

**DOI:** 10.1038/sj.bjc.6602554

**Published:** 2005-04-12

**Authors:** I Willits, L Price, A Parry, M J Tilby, D Ford, S Cholerton, A D J Pearson, A V Boddy

**Affiliations:** 1 Northern Institute for Cancer Research, University of Newcastle, Newcastle upon Tyne NE2 4HH, UK; 2 School of Biomedical Sciences, University of Newcastle, Newcastle upon Tyne NE2 4HH, UK; 3 School of Medical Education Development, University of Newcastle, Newcastle upon Tyne NE2 4HH, UK; 4 School of Clinical Medical Sciences, University of Newcastle, Newcastle upon Tyne NE2 4HH, UK; 5 Paediatric Oncology, Royal Victoria Infirmary, Newcastle upon Tyne, UK

**Keywords:** ifosfamide, comet, DNA damage, paediatric

## Abstract

The degree of damage to DNA following ifosfamide (IFO) treatment may be linked to the therapeutic efficacy. The pharmacokinetics and metabolism of IFO were studied in 19 paediatric patients, mostly with rhabdomyosarcoma or Ewings sarcoma. Ifosfamide was dosed either as a continuous infusion or as fractionated doses over 2 or 3 days. Samples of peripheral blood lymphocytes were obtained during and up to 96 h after treatment, and again prior to the next cycle of chemotherapy. DNA damage was measured using the alkaline COMET assay, and quantified as the percentage of highly damaged cells per sample. Samples were also taken for the determination of IFO and metabolites. Pharmacokinetics and metabolism of IFO were comparable with previous studies. Elevations in DNA damage could be determined in all patients after IFO administration. The degree of damage increased to a peak at 72 h, but had returned to pretreatment values prior to the next dose of chemotherapy. There was a good correlation between area under the curve of IFO and the cumulative percentage of cells with DNA damage (*r*^2^=0.554, *P*=0.004), but only in those patients receiving fractionated dosing. The latter patients had more DNA damage (mean±s.d., 2736±597) than those patients in whom IFO was administered by continuous infusion (1453±730). The COMET assay can be used to quantify DNA damage following IFO therapy. Fractionated dosing causes a greater degree of DNA damage, which may suggest a greater degree of efficacy, with a good correlation between pharmacokinetic and pharmacodynamic data.

Ifosfamide (IFO) is used in the treatment of a variety of paediatric tumours, especially sarcomas, and is usually combined with a number of different agents such as vincristine, actinomycin D or doxorubicin (Carli *et al*, 2003). Although it is considered to be an analogue of cyclophosphamide, IFO appears to have specific activity in some tumour types, for example, rhabdomyosarcoma (Breneman *et al*, 2003). Like cyclophosphamide, IFO requires metabolic activation, mediated by cytochrome *P*450 enzymes (Walker *et al*, 1994), initially forming a 4-hydroxy metabolite, which spontaneously releases the active form – isophosphoramide mustard (IPM). Competing pathways for IFO metabolism result in inactive, dechloroethylated metabolites (2-dechloroethylifosfamide (2DCI) and 3-dechloroethylifosfamide (3DCI)) (Kerbusch *et al*, 2001a). In addition, up to 20% of a dose of IFO can be recovered unchanged in the urine. So, in contrast to cyclophosphamide where 90% of a dose is activated, as much as 70% of a dose of IFO may be eliminated by inactivating pathways. An intermediate on the activation pathway is also subject to metabolic inactivation, aldoifosfamide being further oxidised to an inactive carboxy form by aldehyde dehydrogenases (Dockham *et al*, 1992).

The cytotoxic effect of IFO is thought to be mediated by the reaction of IPM, via unstable aziridine intermediates, with nucleophilic bases of DNA (Sladek, 1988). As a bifunctional alkylating agent, IFO is able to form inter- and intrastrand crosslinks, as well as crosslinks between DNA and protein. Previous studies have shown that the DNA damage caused by IFO in its activated form is detectable by COMET analysis (Johnstone *et al*, 2000). This method, also known as single-cell gel electrophoresis, determines the degree of fragmentation of DNA caused by a particular agent under alkali or neutral conditions (Fairbairn *et al*, 1995; Rojas *et al*, 1999). COMET analysis of the IFO-induced DNA damage has been applied to MCF-7 breast cancer cells *in vitro* and to peripheral blood lymphocytes (PBL) and breast tumour cells from patients (Johnstone *et al*, 2000). Another application of COMET to the analysis of IFO DNA interactions is the detection of crosslink formation, as these retard DNA migration after exposure of PBL to ionising radiation (Hartley *et al*, 1999).

In paediatric patients, the metabolism and pharmacokinetics of IFO have been extensively studied (Boddy *et al*, 1993, 1995b; Silies *et al*, 1998; Kerbusch *et al*, 2001a, 2001b, 2001c; Misiura *et al*, 2003). No direct correlation has been found between pharmacokinetic parameters of drug or metabolites and clinical outcome (Kerbusch *et al*, 2001d). It may, therefore, be appropriate to consider an intermediate measure of IFO action such as the degree of DNA damage in PBL. In the current study, PBL from 19 patients have been collected at various times after IFO administration. Data from COMET analysis has been compared to pharmacokinetic parameters. In addition, COMET analysis of DNA damage *in vitro* has been performed, to investigate the relationship between DNA damage and antiproliferative effect.

## PATIENTS AND METHODS

Ifosfamide and its metabolites were the kind gift of Dr Jürg Pohl, ASTA Medica, Germany. All other reagents were obtained from Sigma, Poole, Dorset, UK, except where otherwise indicated, and were of an appropriate analytical grade.

In all, 19 patients (five female) aged between 2 and 19 years were recruited to the study. In each case, informed written consent was obtained from the patient and/or parent, as appropriate, and the study was approved by the ethical committee of the Newcastle Hospitals Trust. Details of patient characteristics, including the protocol that the patients received, IFO dose and schedule are given in Table 1. Of these patients, eight were studied after a second dose of IFO in order to assess the degree of intersubject variability in DNA damage. Treatment was the same on the two courses studied.

Ifosfamide was administered either as a continuous infusion over 3 days (*n*=8) or as a 3 h infusion every 24 h for 3 days (*n*=5). Three patients were treated with just 2 consecutive days of fractionated dosing and three received 3 consecutive days of IFO, but with a 1 h infusion time. No attempt was made to control concurrent medication, but a record was kept of potential interacting drugs (Table 1).

For those patients receiving a 3 h infusion of IFO, blood samples were taken for the determination of IFO and its metabolites before administration, then at 1.5 and 3 h during the infusion and at 0.5, 1, 2, 4, 6 12 and 24 h after the end of infusion. Patients with a 1 h infusion had a pretreatment and end of infusion samples taken together with the postinfusion samples as for a 3 h infusion. Sampling was repeated for each day of drug administration. For those patients receiving a continuous infusion of IFO, sample times were pretreatment, 3, 6, 12, 18, 24, 36, 48, 60 and 72 h during the infusion. Samples were also taken at 2, 4, 6, 12, 18 and 24 h after the end of the infusion.

Plasma was separated by centrifugation at 1500 **g** and stored frozen at −20°C until subsequent analysis. Ifosfamide, 2DCI, 3DCI and carboxyifosfamide were determined by a validated high-performance thin-layer chromatography method (Boddy and Idle, 1992). A minor metabolite, 4-ketoifosfamide, was detected in some patients, but could not be consistently quantified. Noncompartmental methods were used to estimate pharmacokinetic parameters for parent drug as described previously (Boddy *et al*, 1995b), and the linear trapezoidal rule was used to calculate area under the curve (AUC) for parent drug and metabolites. Area under the curve values for the continuous infusion data relate to all 3 days of administration, including data after the end of infusion.

In addition to samples collected for pharmacokinetic analysis, whole blood samples were taken prior to treatment, and at 3 and 24 h after each dose. A final sample was taken prior to the next course of treatment, that is, 3 weeks after the start of the initial course. The sampling times were based on previous data (Johnstone *et al*, 2000). Patients who were studied on more than one course of treatment had a second set of blood samples taken for pharmacokinetic and COMET analysis.

The COMET assay was performed as described previously (Johnstone *et al*, 2000). Peripheral blood lymphocytes were isolated at the time of blood sampling using Lymphoprep. Storage of PBLs was at −80°C, and analysis was always completed within 1 month of sampling. Stability studies indicated that there was no change in the measurement of DNA damage using the COMET assay during 1 month of sample storage. The degree of DNA damage in a given patient sample or *in vitro* investigation was quantified by measuring the distributed tail moment (TM) in a minimum of 30 cells per slide. Images were analysed using the COMOS image analysis software (Biorad Laboratories, Hertfordshire, UK). The TM data were log normalised and a logarithmic mean and standard deviation (s.d.) determined. The number of highly damaged (HD) cells (log TM more than 2 × s.d. from the mean) per sample was calculated. To estimate the overall degree of DNA damage caused during a cycle of chemotherapy, the AUC of percentage of HD cells (AUCHD) per sample plotted against time was calculated. An internal standard of CCRF-CEM cells that had received a standard dose of radiation was included as a control in each COMET experiment.

In order to provide some background to the clinical studies, the effect of IFO and its active metabolites on the formation of COMET-detectable DNA damage *in vitro* was investigated. CCRF-CEM leukaemia cells were exposed to varying concentrations of the precursor of the active 4-hydroxy metabolite (4-hydroperoxyifosfamide) or IPM for up to 72 h, although these unstable metabolites break down quickly in the medium. Growth inhibition was determined by counting the cells using a Coulter counter and was compared to the degree of DNA damage, as measured by the COMET assay. AUCHD was determined by measuring the percentage of HD cells at times 1, 24, 48 and 72 h after the start of drug exposure.

Statistical analysis was performed using Graphpad Prism, version 3 (Graphpad, San Diego USA). Differences between treatments were analysed using one-way analysis of variance and relationships between pharmacokinetic parameters and DNA damage measurements were analysed by linear regression.

## RESULTS

### Pharmacokinetics and metabolism of IFO

The pharmacokinetic parameters and AUC values for IFO and its metabolites are given in Table 2. As reported previously (Boddy *et al*, 1995b; Kerbusch *et al*, 2001c), apparent clearance is greater in those patients receiving a continuous infusion (median 6.2 l h^−1^ m^−2^) compared to day 1 of fractionated dosing (median 4.1 l h^−1^ m^−2^). However, induction of metabolism is also apparent on day 3 of the fractionated schedule with an increase in median clearance from 4.1 to 6.1 l h^−1^ m^−2^. Correspondingly, the half-life of IFO was longest after a single 3 h infusion, and decreased with repeated dosing or after a continuous infusion. The total AUC of the parent drug was not different in those patients having fractionated dosing over 3 days compared to those receiving a continuous infusion and there was no difference in the AUCs of the metabolites comparing the two groups. Although two patients (Nos. 13 and 17) were being treated with the enzyme-inducing anticonvulsant carbamazepine and four (Nos. 2, 7, 8 and 10) were being treated with fluconazole, which may inhibit IFO metabolism, none of these patients had remarkably high or low clearance values. Similarly, plasma concentrations of metabolites were not markedly different for these patients.

### COMET analysis of DNA damage following IFO administration

There was some variation in the degree of DNA damage present in the pretreatment samples of patients receiving IFO for paediatric malignancies. The median TM for all COMETs within an analysis varied from 2 to 13 (arbitrary units), while the percentage of HD cells varied from 1 to 13% of the total. There was no correlation between the apparent amount of COMET-detectable DNA damage and the number of prior courses of chemotherapy or the age of the patient. The patient with the highest degree of pretreatment damage was patient 16, who was a known cigarette smoker. The effect of cigarette smoking on COMET-detectable DNA damage has been noted previously (Hininger *et al*, 2004).

**Figure 1:**
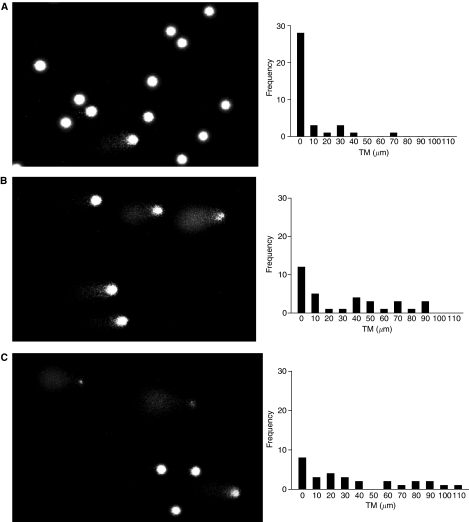

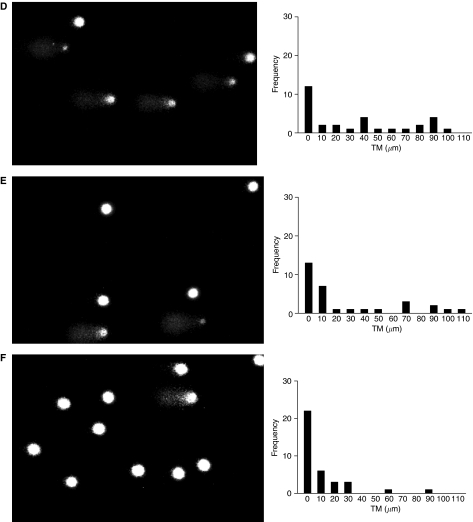
COMETs and histograms of tail moment (TM) for patient 14, course 2. Ifosfamide, 9g m^−2^ fractionated over 3 days, was administered as a 72 h continuous infusion. Pairs of figures show COMETs and histograms (**A**) pretreatment, (**B**) 24 h, (**C**) 48 h, (**D**) 72 h, (**E**) 96 h and (**F**) 3 weeks after the start of infusion.

**Figure 2:**
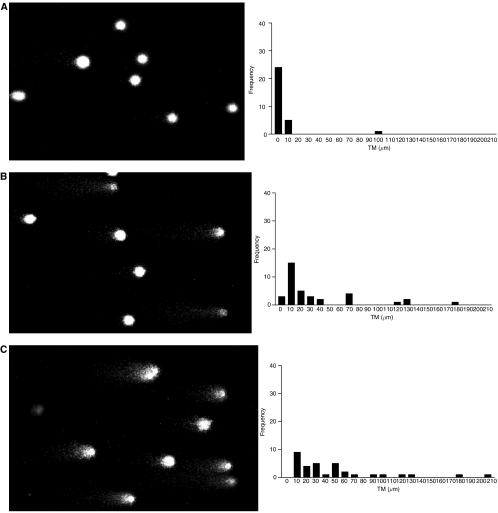

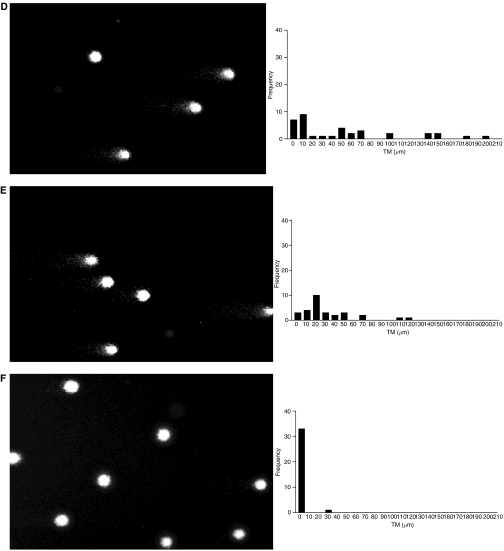
COMETs and histograms of tail moment (TM) for patient 9, course 2. Ifosfamide, 9g m^−2^ fractionated over 3 days, was administered as a 3 h infusion each day. Pairs of figures show COMETs and histograms (**A**) pretreatment, (**B**) 24 h, (**C**) 48 h, (**D**) 72 h, (**E**) 96 h and (**F**) 3 weeks after the start of the first day of administration.

An increase in the degree of damaged DNA was apparent in post-treatment samples from all patients studied (Figures 1A–F and 2A–F). The COMETs and accompanying histograms clearly show the evolution of DNA damage from pretreatment levels to the maximum degree of DNA damage 72 h after the start of administration, for both 72-h infusion and fractionated dosing over 3 days. In both cases, some degree of resolution of damage was apparent 96 h after the start of administration, and DNA damage had returned to pretreatment values prior to the next course of IFO.

**Figure 3:**
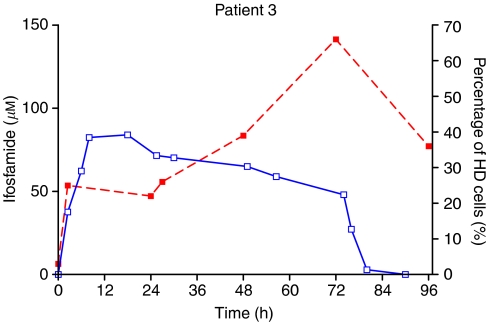
Time course of IFO concentration (solid line) and DNA damage (dotted line) as detected by the COMET assay in patient 3, who was treated with a 72 h continuous infusion. DNA damage quantified as the percentage of damaged cells in PBL samples.

**Figure 4:**
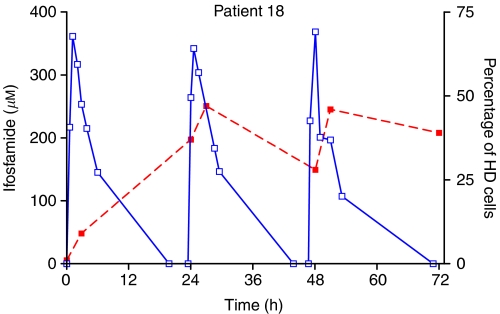
Time course of IFO concentration (solid line) and DNA damage (dotted line) as detected by the COMET assay in patient 18, who was treated with a 3 h infusion on 3 consecutive days. DNA damage quantified as the percentage of damaged cells in PBL samples.

The percentage of HD cells was chosen as a summary parameter for the degree of DNA damage in each patient sample. Analysis based on median TM or other measures of damage did not differ qualitatively from that based on HD. Plots of percent HD against time, superimposed on plasma concentration–time profiles, are shown in Figures 3 and 4 for representative patients who received either a continuous infusion or bolus administration, respectively. Almost all patients showed an accumulation of HD DNA during the time course of IFO administration, although for some patients a degree of resolution between doses on a fractionated regimen was apparent.

**Figure 5:**
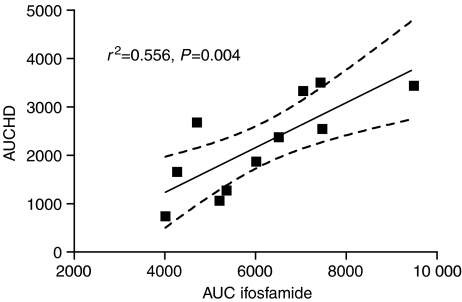
Plot of area under the curve for highly damaged cells (AUCHD) against plasma area under the concentration–time curve for IFO for those patients who received fractionated infusions over 3 days. Line indicates regression, with 95% confidence intervals. AUC for IFO in *μ*M h^−1^. AUCHD units are % × hours.

The relationship between overall DNA damage, as measured by the area under the percentage HD DNA *vs* time curve (AUCHD) and the pharmacokinetics of IFO, was explored. There was a strong positive correlation between the AUCHD and the total AUC of IFO when IFO was administered as a fractionated schedule over 2 or 3 days (Figure 5, *r*^2^=0.556, *P*=0.004). There was also a moderate correlation, in those patients receiving fractionated IFO, between AUCHD and the AUC of the inactive carboxy metabolite (*r*^2^=0.49, *P*=0.023), but there was no other relationship between the pharmacokinetics of IFO and COMET-detectable DNA damage.

**Figure 6:**
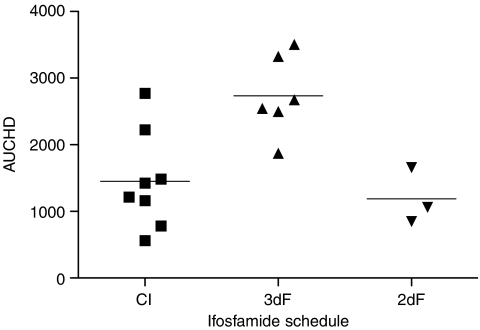
Comparison of summary measure of DNA damage (AUCHD) according to whether IFO was administered at a dose of 3g m^−2^ day^−1^ by continuous infusion over 3 days, by fractionated infusions over 3 days or fractionated infusion over 2 days. AUCHD units are % × hours.

Comparing DNA damage in PBL following IFO administration using either a continuous infusion or 3-day fractionated administration, it is clear that the latter regimen produces a higher AUCHD (Figure 6). The difference between treatment schedules was statistically significant (*P*=0.0035, one-way ANOVA), if the fractionated dosing data are restricted to those patients who received 9g m^−2^, the same dose as the continuous infusion patients.

In those patients studied on more than one occasion, there was good correlation between the AUCHD determined on the two courses (*r*^2^=0.73). Also, there was no significant difference in the DNA damage observed between the two courses, with no indication of cumulative damage or of changes in sensitivity to treatment in successive courses (data not shown).

*In vitro* experiments with CCRF-CEM cells exposed to the active metabolites of IFO (4-hydroxy-IFO and -IPM) showed that the IC_50_ for 4-hydroxy-IFO (19.6±1.5 *μ*M) was much lower than that of IPM (256±39 *μ*M). However, at an IC_50_ concentration, the AUCHD for CCRF-CEM cells treated with either of the two agents were similar, and comparable to those seen in PBL in patients receiving IFO treatment (Table 3). Increasing the concentrations of the active metabolites to 4 × IC_50_ did not significantly elevate the AUCHD.

## DISCUSSION

The role of IFO in treating paediatric malignancies has been the subject of some controversy (Kamen *et al*, 1995; Carli *et al*, 2003). As an alternative to cyclophosphamide, it seems to offer some benefits in terms of antitumour effect, but only at the expense of a wider spectrum of toxicity (Vela-Ojeda *et al*, 2000). While a great deal of research has focused on the metabolism of IFO in relation to side effects such as nephrotoxicity (Boddy *et al*, 1996) and neurotoxicity (Wainer *et al*, 1994), relatively little is known concerning the relationship between systemic pharmacology and the drug interaction with its target, that is, DNA. Understanding of such relationships is complicated by the fact that IFO is a prodrug.

While it would be more relevant to measure the degree of DNA damage in the tumour, this is not practical in most clinical scenarios and there are both ethical and technical problems associated with such measurements. Peripheral blood lymphocytes represent a nonproliferating surrogate tissue; however, PBLs do allow for sequential measurements in the same patient and provide a measure of the systemic exposure to DNA-damaging species. We have previously applied this technique to breast cancer patients being treated with IFO, but had only limited success applying COMET analysis to tumour cells in fine-needle aspirates (Johnstone *et al*, 2000). The COMET method used here detects DNA strand breaks, presumably the result of repair processes or resulting from denaturing of DNA during the method itself (Rojas *et al*, 1999). Ifosfamide is thought to act by forming interstrand crosslinks in DNA and a method has been developed to use the COMET assay to measure such crosslinks directly (Hartley *et al*, 1999). That method was not applied here as it was not available at the start of the analysis and initial attempts to apply the method to the clinical samples were not successful (data not shown).

The pharmacokinetics and metabolism of IFO determined in the 19 patients studied here are very similar to previous reports in paediatric patients (Boddy *et al*, 1993, 1995b; Boos *et al*, 1995; Kerbusch *et al*, 2001b). As has been reported previously, there were no systematic differences between patients treated with a continuous infusion compared to fractionated dosing (Boddy *et al*, 1995b; Boos *et al*, 1995). Induction of metabolism, detected as a decrease in plasma IFO concentration during infusion or on successive days of treatment, was apparent in most patients. This is accounted for largely by an increase in metabolism to inactive dechloroethylated metabolites (Boddy *et al*, 1995a; Kerbusch *et al*, 2001b, 2001c).

The distribution of TMs seen in the pretreatment PBL samples indicated that the method used was selective for DNA damage caused by the administration of chemotherapy. One patient, a 19-year-old smoker, had a slightly shifted distribution of TMs and a greater number of detectable HD cells. However, increases above background in both TM distribution and percentage HD were seen in this patient, as with the other patients studied. Figures 1 and 2 show the evolution of DNA damage, as detected by the COMET assay, before, during and after IFO administration and Figures 3 and 4 overlay the DNA damage data with plasma concentration–time curves for individual patients.

In order to understand the relationship between DNA damage and the systemic pharmacology of IFO, a summary measure of DNA damage AUCHD was plotted against pharmacokinetic parameters for IFO and AUC values for its metabolites. The only significant correlation was found between AUCHD and AUC for IFO itself, and then only for those patients where IFO was administered as fractionated infusions over 3 days. This is in contrast to previous reports on cyclophosphamide, where either antitumour effect or toxicity has been found to be inversely related to plasma AUC for the parent drug (or positively correlated to clearance) (Ayash *et al*, 1992; Yule *et al*, 2004). This discrepancy in the data for these two related drugs, which are thought to share a common mechanism of action, may relate to the differing contributions of activating and inactivating pathways of metabolism (Boddy and Yule, 2000). Cyclophosphamide elimination is largely due to metabolism via the initial 4-hydroxylation of the oxazaphosphorine ring. Only 5% or less of the metabolism of the parent drug is via the inactivating dechloroethylation pathway (Moore, 1991). In contrast, 50% of the metabolism of IFO is by dechloroethylation and up to 23% of a dose is eliminated unchanged in the urine (Kerbusch *et al*, 2001a), suggesting that elimination of parent drug reflects inactivation for IFO. We have previously reported a weak correlation between DNA damage and plasma concentrations of the 4-hydroxy metabolite of IFO, although this unstable metabolite was only measurable in four patients (Johnstone *et al*, 2000). The fact that a weak correlation was seen in the present study between AUCHD and AUC for carboxy-IFO, which is formed by sequential metabolism of 4-hydroxy-IFO, may suggest that detection of the carboxy metabolite in plasma largely reflects activation of the parent drug.

Comparisons of IFO administered either as a 72 h continuous infusion or as a short infusion on 3 consecutive days showed no consistent difference in the either the metabolism or the clearance of the parent drug (Boddy *et al*, 1995b; Boos *et al*, 1995). Data from the study reported here, without the within-subject crossover of previous studies, again indicated no difference in the systemic pharmacology of IFO between the two schedules. However, there was a significant difference between the two schedules of administration with regard to the degree of damage in PBL. The mean AUCHD was 2497 for the fractionated dosing over 3 days, whereas that seen following the continuous infusion was 1453. A lower AUCHD (mean 1190) was observed following 2 days of fractionated dosing. This difference in pharmacological effect between the two schedules suggests that the fractionated dosing schedule may be more effective, perhaps with a greater risk of toxicity. The mechanism underlying this difference may relate to changes in enzyme levels during continuous exposure to parent drug, or might reflect changes in the activity of activating enzymes following exposure to toxic metabolites. There were insufficient numbers of patients with consistent treatment in this study to analyse any relationship to toxicity. Values of AUCHD observed in PBL in patients were similar to those associated with growth inhibition *in vitro* in CCRF-CEM cells.

The data reported here on DNA damage following IFO administration indicate, in a very heterogeneous patient population, that the COMET assay may provide useful information on the pharmacology of IFO. Fractionating the dose of IFO over 3 days appears to result in a greater degree of DNA damage than continuous infusion of the same dose. Also, for patients receiving IFO as a fractionated schedule, there was a direct correlation between overall DNA damage and the AUC of the parent drug. Further studies with patient groups receiving more uniform treatment should focus on the relationship between DNA damage and clinical outcome, and also incorporate measures of DNA crosslink formation (Hartley *et al*, 1999), in addition to the strand breaks as detected here. Clinical use of COMET analysis in PBLs requires further validation by comparison with tumour DNA damage or clinical outcome.

## Figures and Tables

**Table 1:** Patient details

**Patient number**	**Sex**	**Age (years)**	**Diagnosis**	**Course studied**	**Course type**	**Ifosfamide dose (g m^−2^)**	**Other medication**
1	M	2	RM	3	IE_1_	9	Imi, ond, tei
2	F	3	RM	4	IE_1_	9	acy, cot, **flu**, fru, ond
3	M	10	HE	1	IE_1_	9	Cot, ond, par
4	M	17	RM	1	IE_1_	9	Chl, dex, gav, fru, ond, par, sen, tem
5	M	5	RM	5	IVA_1_	9	Cep, chh, dex, lac, tri
6	F	2	RM	3	IVA_1_	9	Chh, dex, ond, tri
7	M	19	MFH	1	IE_1_	9	Dih, **flu**, fru, imi, lac, nab, ond
8	F	15	ES	4	IVA_3_	6	Dex, **flu**, lac, mor, nab, ond, ran
9	M	4	RM	2	IVA_1_	9	Dex, ond
10	M	19	PNT	4	IE_1_	9	Ami, cod, cyc, dex, **flu**, gav, imi, met, **ran**, sen, sep, tei, tem
11	M	6	RM	4	IVA_2_	6	Cep, fru, ond, lor, mtp, ond
12	F	3	RM	3, 6	IVE	9	Dex, ond
13	M	19	RM	5, 9	IVE	9	Ami, car, cod, dex, met, ond mbl cla
14	M	19	OS	2, 3	IE_2_	9	Dex, lac, met, ond
15	M	15	OS	2, 4	IE_2_	9	Dex, met, ond
16	M	19	ES	3, 5	IVAd	6	Cyc, dex, met, ond
17	M	7	RM	2, 4	IVA_2_	6	Car, cod, cyc, lac, ond
18	M	10	ES	2, 4	IVA_3_	6	Dex, ond, cyc, dex, lor, met
19	F	3	RM	3, 4	IVA_2_	6	Dex, met, ond, ran, par

*Diagnosis*: ES=Ewings sarcoma; HE=haemangioendothelioma; MFH=malignant fibrous histiocytoma; PNT=primitive neuroectodermal tumour; OS=osteogenic sarcoma; RM=rhabdomyosarcoma.

*Course type*: IE_1_ – ifosfamide 3 g m^−2^ day^−1^ continuous infusion over 3 days. Etoposide 200 mg m^−2^ day^−1^ as a 2 h infusion on 3 consecutive days. IE_2_ – as IE_1_, etoposide 150 mg m^−2^ day^−1^. IVA_1_ – ifosfamide 3g m^−2^ day^−1^ as a 3 h infusion on 3 consecutive days. Actinomycin D and vincristine both 1.5 mg m^−2^ bolus on day 1 only. IVA_2_ – as IVA_1_, ifosfamide on 2 days only. IVA_3_ – as IVA_1_, ifosfamide 2g m^−2^ day^−1^ 1 h infusion on 3 consecutive days. IVAd – as IVA_3_, but adriamycin 20 mg m^−2^ day^−1^ as a 6 h infusion on 3 consecutive days, no actinomycin D. IVE – ifosfamide 3g m^−2^ day^−1^ as a 3 h infusion, etoposide 150 mg m^−2^ as a 4 h infusion, each on 3 consecutive days. Vincristine 1.5 mg m^−2^ bolus on day 1 only.

*Other medication (drugs taken during study)*: Acy=acyclovir; amy=amitriptyline; car=carbamazepine; cep=cephalexin; chh=chloral hydrate; chl=chlorpromazine; cla=clarithromycin; cod=codeine phosphate; cot=cotrimoxazole; cyc=cyclozine; dex=dexamethasone; dih=dihydrocodeine; dox=doxorubicin; flu=fluconazole; fru=frusemide; gav=gaviscon; imi=imipenem; lac=lactulose; lar=lorazepam; mbl=methlyene blue; met=metoclopropamide; mtp=methotrimeprazine; mor=morphine sulphate; nab=nabilone; ond=ondansetron; par=paracetamol; ran=ranitidine; sen=senna; tei=teicoplanin; tem=temazepam; tri=trimeprazine. Drugs thought to inhibit CYP3A4 are shown in bold, drugs known to induce CYP3A4 are underlined. All patients were also hydrated with mesna at an equivalent dose to ifosfamide (2 or 3 g m^−2^ day^−1^ in 3 l). This was administered as a continuous infusion throughout the course and for 12 h after completion of the course.

**Table 2:** Pharmacokinetics and metabolism of ifosfamide in the patients studied

		**IFO PK**			**Metabolite AUCs (*μ*M h)**			
**Regimen**	***N***	**Clearance (l h^−1^ m^−2^)**	***t*_1/2_ (h)**	***V*_d_ (l kg^−1^)**	**IFO**	**CXI**	**3DCI**	**2DCI**
72 h CI	8	6.2 (2.6–7.4)	2.4 (1.3–5.6)	0.44 (0.16–1.82)	5135 (4668–13247)	941 (542–3443)	1868 (554–5342)	1166 (207–3011)
3 or 1 h infusion day 1	11	4.1 (2.6–5.8)	4.7 (1.9–8.3)	0.89 (0.35–1.63)	6011 (4009–9488)	511 (47–1700)	1702 (521–3164)	1337 (326–2678)
3 or 1 h infusion day 3	8	6.1 (3.0–7.8)	2.9 (2.0–4.6)	0.92 (0.53–1.33)				

IFO-PK=pharmacokinetic parameters for ifosfamide; *t*_1/2_=half-life; *V*_d_=volume of distribution.

For metabolite abbreviations see text.

AUCs for IFO and metabolites in patients receiving a fractionated dosing over 3 days are given cumulatively over the cycle of chemotherapy to allow for comparison with data from patients receiving the same dose as a continuous 72 h infusion (CI). AUCs are dose adjusted in those patients receiving only 2 days of therapy. Data have been combined for those patients receiving fractionated doses of ifosfamide by either 1 or 3 h infusion.

**Table 3:** AUCHD for CCR-CEM cells treated with IPM or 4-hydroxy-IFO *in vitro*

	**Treatment**	**AUCHD**
*In vitro*	IPM IC_50_	1399±147
	IPM 4 × IC_50_	2549±395
	4-hydroxy-IFO IC_50_	2850±407
	4-hydroxy-IFO 4 × IC_50_	3185±819
		
Clinical data	IFO continuous infusion	1453±730
	IFO 3 × daily infusion	2736±597
	IFO 2 × daily infusion	1190±419

AUCHD=area under the curve of % highly damaged cells; IPM=isophosphoramide mustard.

For comparison, AUCHD data are also shown for patients treated with either continuous infusion or fractionated dosing of ifosfamide. AUCHD given as mean±s.d. Units are % × hours.
